# Lymphadenitis caused by *Legionella sainthelensi* infection: a case report and literature review

**DOI:** 10.3389/fmed.2025.1574205

**Published:** 2025-05-20

**Authors:** Cangjian Zhang, Minlei Zhao

**Affiliations:** Department of Hematology, Lishui Municipal Central Hospital, Lishui, China

**Keywords:** lymphadenitis, *Legionella sainthelensi*., infectious diseases, next-generation sequencing, fever

## Abstract

**Background:**

Legionella infection is a group of bacterial diseases caused by species of the Legionella genus, the most notable of them being Legionnaires’ disease and Pontiac fever. Legionnaires’ disease primarily affects the lungs, with the respiratory and gastrointestinal systems being the most commonly affected. Legionella may cause multisystem involvement, according to this study. In this report, we present a rare case of lymphadenitis caused by *Legionella sainthelensi*, aiming to raise awareness of atypical manifestations and uncommon sites of Legionella infection.

**Case presentation:**

We present the case of a 43-year-old man who was admitted to the hospital for a week due to fever and enlargement of the cervical lymph nodes. He was diagnosed with *L. sainthelensi* lymphadenitis, which was treated with azithromycin and lymph node puncture and drainage.

**Conclusion:**

For patients with Legionella lymphadenitis, multiple etiological tests should be performed as early as possible to confirm the infectious pathogen. Early intervention with appropriate intravenous antibiotics and lymph node drainage is critical to improve the cure rate.

## Introduction

Legionella is a facultative intracellular pathogen that widely exists in many different natural and artificial aquatic environments and can infect human monocytes and macrophages, resulting in severe pneumonia known as Legionnaires’ disease. Nearly half of the more than 60 different species in the Legionella genus have been related to human diseases (1, 2). Symptoms result from inhalation of Legionella-contaminated aerosols through the respiratory tract, followed by bacterial replication within alveolar macrophages, resulting in two types of distinct clinical presentations: Pontiac fever, a self-limited flu-like illness, and Legionnaires’ disease, an atypical pneumonia caused by an acute lower respiratory tract infection (3). *Legionella pneumophila* and *Legionella longbeachae* are common Legionnaires’ disease pathogens reported in the clinic (4).

We report the first documented case of *L. sainthelensi* lymphadenitis, a rare infection of the lymph nodes.

## Case presentation

**Figure 1:**
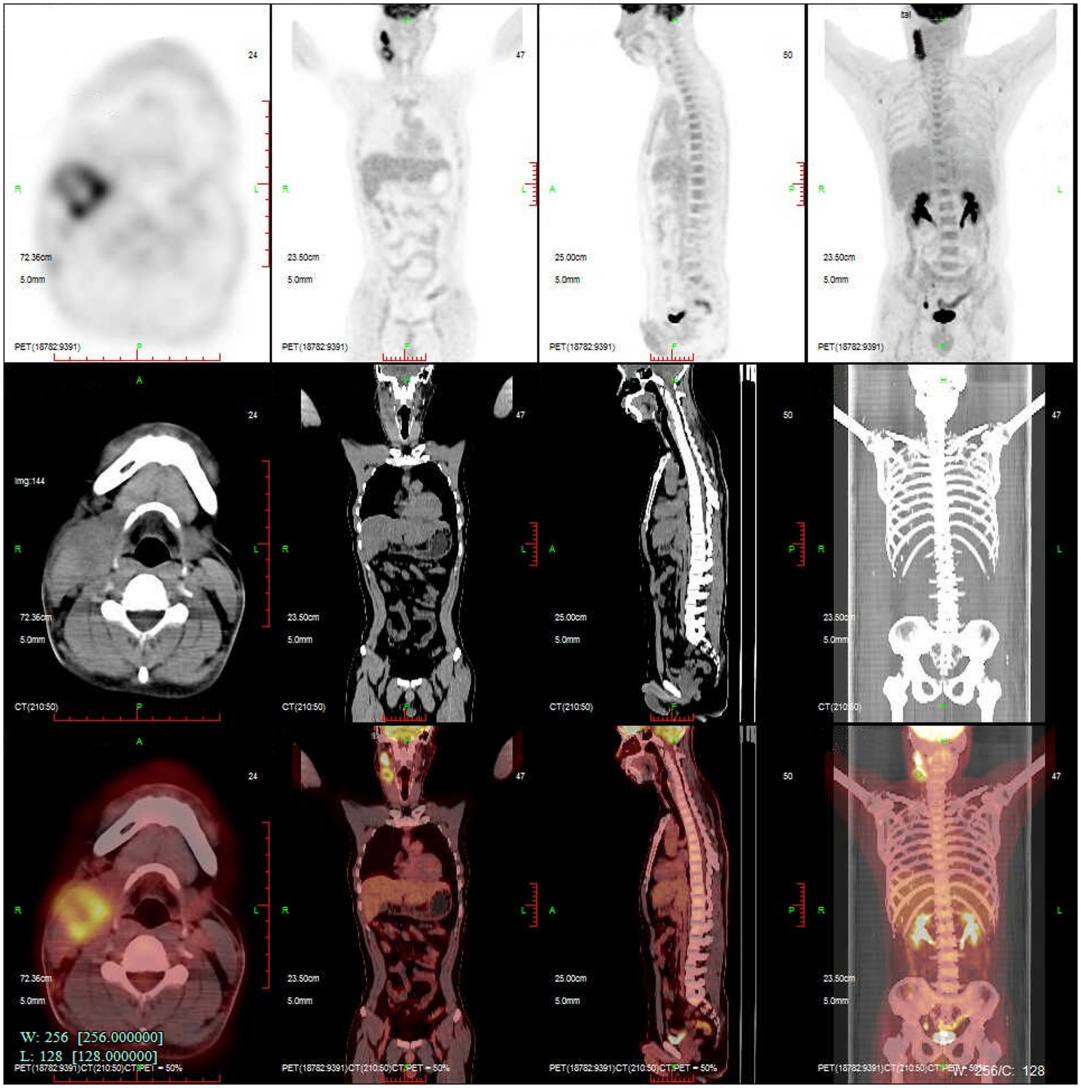
PETCT reveals several enlarged lymph nodes in the right neck with abnormally increased FDG metabolism. Malignant tumor is suspected.

**Figure 2:**
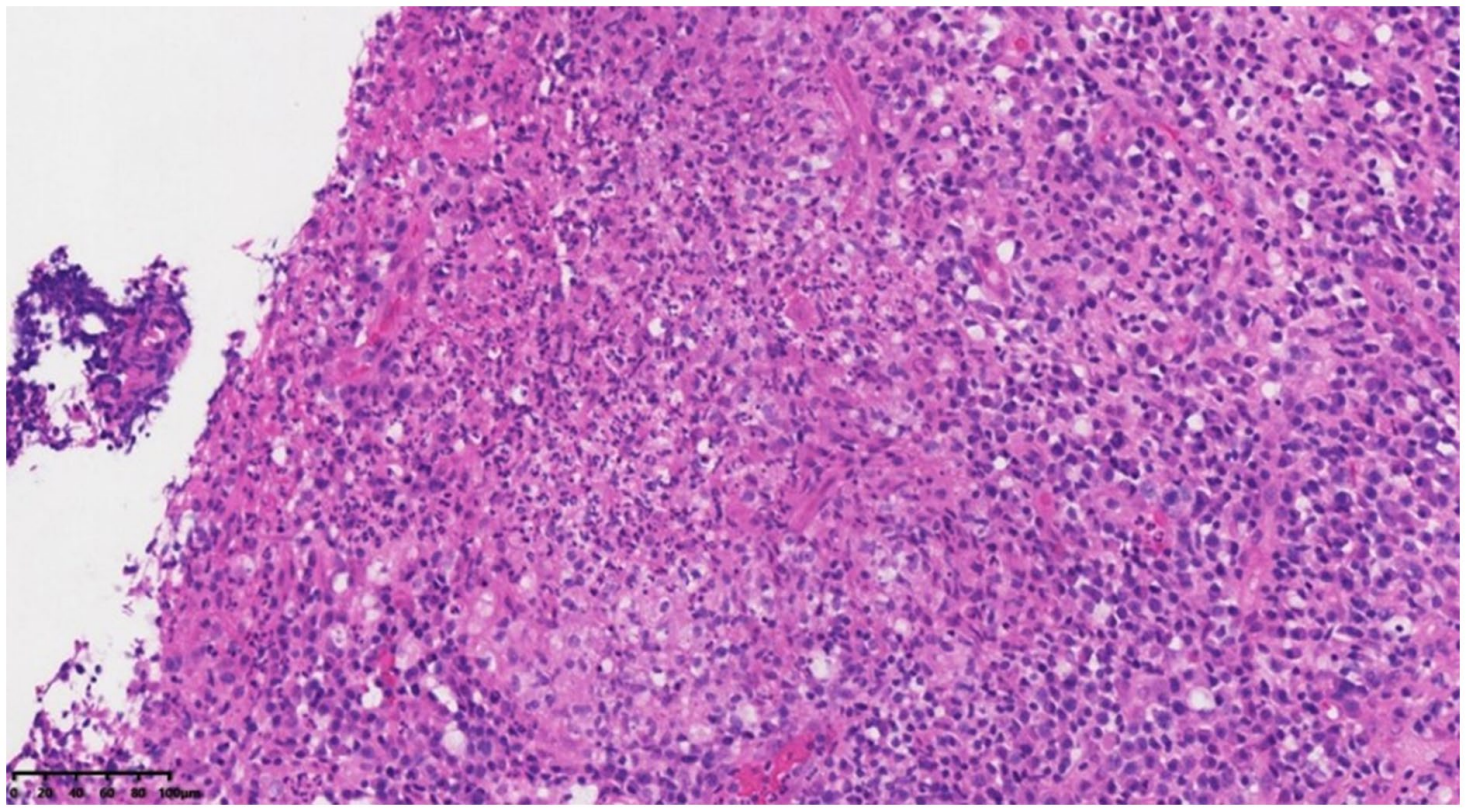
Lymph node structure was disordered, local neutrophil infiltration was accompanied by microabscess formation, and the surrounding tissue cells were aggregated to form granulomatous changes of HEX200.

**Figure 3:**
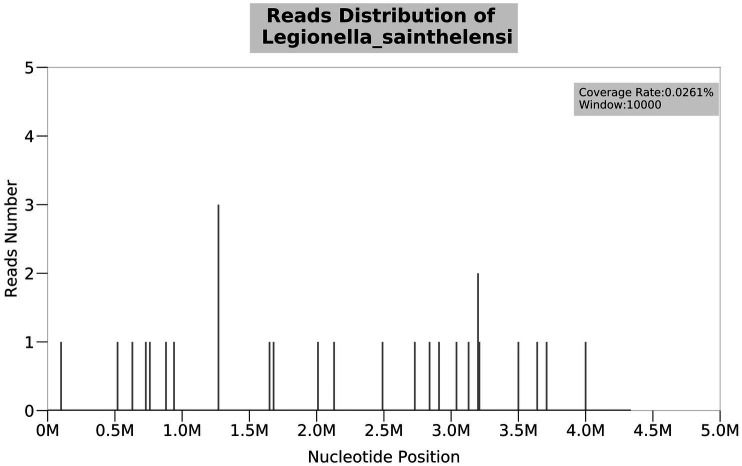
NGS test result of the puncture fluid shows *Legionella sainthelensi*. The number of detected sequences is 18, and the relative abundance is 25.17%.

On 28 November 2022, a 43-year-old man was admitted to the hospital after complaining of fever and enlargement of cervical lymph nodes for 1 week. He was a glass factory worker who lived in a rural area and had a history of contact with stagnant water. The patient presented with enlargement of cervical lymph nodes accompanied by pain and fever that began 1 week prior without an obvious cause. He was admitted to a local hospital on 25 November 2022. A B-mode ultrasound scan of the neck revealed enlargement of the right cervical lymph nodes accompanied by partial liquefaction. Despite treatment with cefuroxime, the symptoms did not improve significantly. The patient presented with fever and neck lymph node pain without cough or chest tightness. For further treatment, the outpatient department intended to admit the patient for cervical lymph node enlargement. It is recommended that patients with a history of hypertension take nifedipine sustained-release tablets 30 mg QD orally and valsartan tablets 80 mg QD orally to lower blood pressure. On 29 November 2022, post-admission abnormal laboratory values included CRP 48.57 mg/L (<8), leukocytes 11.7 × 109/L (3.5–9.5), hemoglobin 151 g/L (130–175), platelets 345 × 10^9^/L (125–350), procalcitonin 0.10 ng/mL (<0.05), and fibrinogen 6.20 g/L (2.4–4.0). Liver and kidney functions were normal, and T-SPOT negativity was observed. PET-CT (Figure 1) images on 1 December 2022 revealed the following: (1) several swollen lymph nodes in the right neck, an abnormal increase in FDG metabolism, the possibility of malignant tumor, and the need for a high metabolic lesion biopsy and (2) the possibility of right axillary lymph node hyperplasia in the right supraclavicular region. After admission, the patient underwent an ultrasound-guided mass puncture examination. The puncture fluid was pale yellow. The bacterial culture of the puncture fluid was negative for acid-fast bacilli. Pathological results of the right cervical lymph node puncture revealed lymph node structure disorder, local infiltration of a large number of neutrophils with pus formation, surrounding tissue cell aggregation to form granulomatous changes, and interstitial fibrous tissue hyperplasia with a large amount of plasma cell infiltration. Combined with the immunohistochemistry results, inflammatory lesions were considered. The immunohistochemical labeling results revealed CD3 + and CD5 + T cells, CD20 + and PAX5 + B cells, activated CD30 + cells, CD68 + tissue cells, FDC network CD21 positivity, and Ki-67 positivity (T zone increased). Molecular *in situ* hybridization revealed EBER negativity (Figure 2). Subsequently, ultrasound-guided lymph node puncture and abscess drainage catheterization were performed again. The results of next-generation sequencing (NGS) revealed *L. sainthelensi*, with a detected sequence number of 18 and a relative abundance of 25.17% (Figure 3). The urine antigen test (UAT) for Legionella was weakly positive, supporting the diagnosis of *L. sainthelensi* lymphadenitis. Later, the patient was treated with azithromycin injection at a dose of 0.5 g once a day for intravenous drip treatment. After 2 weeks, the swelling in the neck decreased significantly. Upon discharge, the patient was prescribed azithromycin tablets at a dose of 0.5 g once a day for oral administration for another 2 weeks. At the 1 month follow-up visit, the patient returned to our hospital and the lymph node abscess was completely absorbed.

## Discussion

Legionnaires’ disease is an infectious disease that has a high recessive infection rate and a high mortality rate that poses a serious threat to people’s health. In recent years, Legionella has had a high incidence associated with hospitalization in the intensive care unit. The incidence of Legionellosis in the United States is approximately 1.8 per 100,000 population per year, although underreporting is common. Early detection and diagnosis are particularly important to reduce the mortality rate (5). Since the Philadelphia outbreak in 1976, the number of recognized species and serogroups of Legionella has continued to increase, with more than 60 species and 80 serogroups being discovered. *L. sainthelensi* was first identified in freshwater cultured near Mount St. Helens in 1981 (6). We report the first case of *L. sainthelensi* infection in the lymph nodes. To date, 15 cases of *L. sainthelensi* infection have been reported worldwide (Table 1) (7–11). Other reports have focused on lung-related infections, but this is the first report of an *L. sainthelensi* lymph node infection.

**Table 1:** *L. sainthelensi* infection have been reported worldwide.

References	Year	Number of case(s)	Age	Sex	Type of sample	Technique of identification	Antibiotic therapy	Death
Benson et al. (7)	1989	3			Pleural fluid	Culture on specific agar	Erythromycin	Yes
Han et al. (9)	1994	9	69–102		Serm	Serologic testing antibody		
Loeb et al. (8)	2015	1	28		Broncho alveolar lavage	Direct fluorescent antibody assay	Ceftriaxone, azithromycin, gatifloxacin	No
Slow et al. (10)	2001	1				Culture on specific agar		
Kamus et al. (11)	2021	1	35			PCR		No

Normally, Legionella infection can be diagnosed based on a variety of tests, including the UAT, specimen culture, nucleic acid tests, and serum antibody tests. In traditional laboratory detection methods, bacterial isolation and culture methods are the “gold standard” for the identification of Legionella infection in the laboratory for diagnosis and epidemiological investigation. Legionella, however, is a fastidious bacterium with special nutritional requirements. Legionella grows slowly and is easily outcompeted by other miscellaneous bacteria during growth. Due to its growth characteristics, Legionella has high requirements for specimen collection quality and operation technology, strict culture nutrient conditions, and a long time for growth. Although the culture method has good specificity (100%), its sensitivity is low (50–80%), and the positive detection rate in China is generally low (12). Serum antibody detection of Legionella is widely used in our country, and it has diagnostic significance when double serum samples from the acute and convalescent stages show four or more changes. Most patients with Legionella infections develop antibodies by the third week of infection. Edelstein et al. (13) revealed that approximately 25% of patients with Legionella infection confirmed by etiological culture did not show elevated serum antibodies and that immunosuppressed patients may not produce serum antibodies permanently. The detection of the serum antibody titer of Legionella has certain value for epidemiological investigation and retrospective analysis, but it is not of high value for early diagnosis. The UAT is widely used in Europe, America, and Japan; at present, 82 and 97% of Legionnaires’ disease cases in the United States and the European Union are diagnosed by the UAT (14). UAT detects lipopolysaccharide in the Legionella cell wall. Urine antigen was detected to be positive 2–3 days after infection and negative 2–3 months after treatment (15). In recent years, molecular biological detection and identification technology based on the polymerase chain reaction (PCR) has been widely used in the detection, identification, and typing of Legionella. Compared to the traditional culture method, PCR-based detection is faster, can detect dead Legionella and living non-cultivable Legionella, and has high sensitivity and specificity. A multicenter study in Belgium compared the sensitivity of PCR and UAT detection of Legionella, and the results showed that the sensitivity of the PCR method (64.6%) was significantly higher than that of UAT (44.4%) (16).

NGS is a nucleic acid detection technology that has emerged in recent years, and it can be used to directly perform untargeted high-throughput sequencing of the genomes of all pathogens in clinical samples (17). NGS is highly sensitive and has low requirements for pathogen load in clinical samples. NGS can simultaneously identify multiple pathogens and accurately and efficiently retrieve nucleic acid information for all pathogens in samples, helping clinicians quickly identify pathogenic pathogens. Specifically, it has advantages in the detection of fastidious bacteria, slow-growing bacteria, pathogens that cannot be isolated and cultured, and pathogens of emerging infectious diseases, which can assist in the diagnosis of pathogens of infectious diseases (18). During an epidemiological study of Legionella infection in Europe, the sensitivity and specificity of using the STB gene for detection were significantly increased by amplifying and sequencing the STB gene. This strategy was gradually applied to clinical diagnostic detection, contributing to the rapid development of mNGS (19). It has been reported that mNGS can improve the detection rate of Legionella (20, 21). In this study, the patient was admitted to the hospital for lymph node enlargement accompanied by fever. After B-mode ultrasound-guided puncture, pathological results indicated inflammatory changes, and bacterial culture of the puncture fluid and acid-fast bacilli testing were negative. NGS was performed, and *L. sainthelensi* was detected. The Legionella UAT was weakly positive, and *L. sainthelensi* infection in the lymph nodes was confirmed.

Legionella is an intracellular bacterium; thus, effective anti-infective drugs against Legionella depend on anti-Legionella drug activities and concentrations in alveolar macrophages. β-lactam and aminoglycoside antibiotics are not effective in the treatment of Legionella pneumonia due to their inability to permeate the cell membrane. Empiric therapies for Legionella infection include quinolones, macrolides, doxycycline, tigecycline, cotrimoxazole, and rifampicin, all of which have been confirmed to be effective in the treatment of Legionella pneumonia. Since the severity of Legionella pneumonia is closely related to the bacterial load and the host’s immune status, it is essential to make accurate diagnoses and provide appropriate anti-infective treatments and interventions at an early stage (22). At present, the guidelines available for treatment of Legionella worldwide are primarily for Legionella pneumonia; however, there are no treatment guidelines for Legionella lymphadenitis. The recommended duration of treatment for Legionella pneumonia is 2 weeks for the immune normal host and 3 weeks for the immunosuppressed host. A shorter course of treatment may lead to recurrence (23, 24). In this study, the patient underwent lymph node puncture followed by the insertion of a drainage tube for the implementation of negative-pressure pus drainage. Azithromycin was administered for anti-infectious therapy. During the 2 weeks of treatment, a significant reduction and absorption of the mass were observed. Subsequently, the patient was discharged from the hospital and transitioned to a 2-week regimen of oral medication for consolidation treatment. The patient was then discharged home with oral antibiotic consolidation therapy for 2 weeks. In patients with Legionella lymphadenitis, pus may need to be drained in addition to anti-infective drug therapy to achieve better clinical efficacy.

## Conclusion

In conclusion, the incidence of Legionella infection in lymph nodes is extremely low. Multiple pathogen tests should be performed on suspected patients as early as possible, and early diagnosis and appropriate anti-Legionella treatment are key to improving the cure rate of the treatment and reducing the mortality rate.

## Data Availability

The original contributions presented in the study are included in the article/supplementary material, further inquiries can be directed to the corresponding author.
